# Host range and community structure of avian nest parasites in the genus *Philornis* (Diptera: Muscidae) on the island of Trinidad

**DOI:** 10.1002/ece3.1621

**Published:** 2015-08-15

**Authors:** Mariana Bulgarella, George E Heimpel

**Affiliations:** Department of Entomology, University of MinnesotaSt. Paul, Minnesota, 55108

**Keywords:** Bird–parasite interactions, community similarity, host specificity, Jaccard index, *Philornis*, Trinidad

## Abstract

Parasite host range can be influenced by physiological, behavioral, and ecological factors. Combining data sets on host–parasite associations with phylogenetic information of the hosts and the parasites involved can generate evolutionary hypotheses about the selective forces shaping host range. Here, we analyzed associations between the nest-parasitic flies in the genus *Philornis* and their host birds on Trinidad. Four of ten *Philornis* species were only reared from one species of bird. Of the parasite species with more than one host bird species, *P. falsificus* was the least specific and *P. deceptivus* the most specific attacking only Passeriformes. *Philornis* flies in Trinidad thus include both specialists and generalists*,* with varying degrees of specificity within the generalists. We used three quantities to more formally compare the host range of *Philornis* flies: the number of bird species attacked by each species of *Philornis*, a phylogenetically informed host specificity index (Poulin and Mouillot's *S*_TD_), and a branch length-based *S*_TD_. We then assessed the phylogenetic signal of these measures of host range for 29 bird species. None of these measures showed significant phylogenetic signal, suggesting that clades of *Philornis* did not differ significantly in their ability to exploit hosts. We also calculated two quantities of parasite species load for the birds – the parasite species richness, and a variant of the *S*_TD_ index based on nodes rather than on taxonomic levels – and assessed the signal of these measures on the bird phylogeny. We did not find significant phylogenetic signal for the parasite species load or the node-based *S*_TD_ index. Finally, we calculated the parasite associations for all bird pairs using the Jaccard index and regressed these similarity values against the number of nodes in the phylogeny separating bird pairs. This analysis showed that *Philornis* on Trinidad tend to feed on closely related bird species more often than expected by chance.

## Introduction

One of the most fundamental characteristics of a parasite is the spectrum of host species that it can exploit (Adamson and Caira 1994; Poulin 2007). Interactions between birds and their arthropod parasites illustrate the broad range of host specificity that is possible, ranging from parasites with very broad host ranges such as the hen flea, *Ceratophyllus gallinae*, which attacks at least 72 host species (Tripet and Richner 1997), to some feather lice species that only attack one species or strain of host (Clayton 1991; Johnson et al. 2002). Bird species can also vary greatly in the number of parasite lineages that they support (e.g., Lacorte et al. 2013), suggesting differences in the host resistance profile or parasite pressure. Here, we take a phylogenetic approach in analyzing host range, parasite species load, and community similarity in associations between a genus of parasitic flies and their bird hosts in Trinidad.

One way of quantifying the host range of a parasite is to simply count the number of hosts it can exploit. However, such estimation lacks important information about the phylogenetic relationship among the host species. Poulin and Mouillot (2003) developed a host specificity index, *S*_TD_, that measures the standardized taxonomic distinctiveness of all host species used by a parasite. *S*_TD_ allows for the differentiation of parasite species that attack the same numbers of host species at different levels of phylogenetic organization. For example, a parasite species attacking two host species in the same genus has a lower *S*_TD_ value than a parasite species attacking two host species in different genera.

The number of parasite species supported by single host species (the ‘parasite species load’) is another important aspect of host–parasite interactions and can reflect processes such as host susceptibility and parasite competition (Godfray 1994). Here again a phylogenetic approach can be illuminating. In particular, determining the signal of parasite species load on the phylogeny of a group of hosts could be used to pose hypotheses concerning the evolution of susceptibility or resistance in host clades, and of niche partitioning among parasite species (Poulin 2007).

Finally, integrating information on the community structure of parasites and their hosts with information on host phylogenies has the potential to improve our understanding of the patterns and determinants of host range. Recently, indices of species diversity have been applied to calculate how similar the parasite faunas of host species are (Novotny et al. 2002; Weiblen et al. 2006; Vinarski et al. 2007; Davies and Pedersen 2008; Poulin 2010), driving a new interest in the understanding of community similarity of host species, also known as faunal similarity. The Jaccard index is widely used to assess community similarity across geography and phylogeny, and for the case of parasites, a number of studies have shown that the similarity in species composition decreases exponentially with phylogenetic and geographic distance among the host species (Poulin 2003, 2010; Fellis and Esch 2005; Krasnov et al. 2005; Oliva and González 2005; Poulin et al. 2011). At the phylogenetic level, this negative relationship is expected because closely related hosts tend to have similar physiologies and resistance, susceptibility, or tolerance profiles (e.g., Desneux et al. 2012). Therefore, closely related host species are expected to harbor more similar parasite faunas than are distantly related ones (Poulin 2010).

Host range, parasite species load, and community similarity are important descriptors of host–parasite interactions. Few studies have used a phylogenetic approach to analyze all three of these processes in a community of bird parasites and their hosts. In this article, we follow such an approach using *Philornis* parasites on the island of Trinidad.

## Materials and Methods

### Study system

We studied the associations between the avian-parasitic flies in the genus *Philornis* (Diptera: Muscidae) and their host birds. In particular, we analyzed data compiled by Dodge and Aitken (1968) over 6 years of fieldwork (1956–1961) for ten *Philornis* species parasitizing 29 bird species belonging to fourteen families on the island of Trinidad. While conducting arthropod-borne virus research on this island, Dr. Aitken collected large quantities of *Philornis* material. He reared the adult flies from larvae or pupae taken from the bird nests. The taxonomic classification of the 29 species of birds and the type of nest they use can be found in Table S1.

The distribution of the genus *Philornis* is Neotropical being found from Argentina and Chile northwards to Texas and Florida, USA (Skidmore 1985), with fifty species currently recognized (Couri et al. 2007). *Philornis* species depend on birds to complete their life cycle as the larval stage is parasitic. According to the habits of their larvae, they are divided into three feeding guilds. The majority of *Philornis* species with known larval habits (currently 28 species) are subcutaneous blood feeders, two species are coprophagous with free-living larvae in the nest, and two species have free-living, semi-hematophagous larvae (Teixeira 1999). A list of the ten *Philornis* species that occur on Trinidad, their larval habits, and taxonomic synonymies is presented in Table1.

**Table 1:** The ten species of *Philornis* occurring in Trinidad, with synonymies, larval habits, number of host birds exploited, the *S*_TD_ index, and the branch length-based *S*_TD_

*Philornis* species	Synonymies	Larval habit	Number of host birds	*S*_TD_	Branch length-based *S*_TD_
*P. aitkeni*		Free-living coprophagous	1	1	0
*P. falsificus*	*P. falsifica*	Free-living semi-hematophagous	3	4.0	163.5
*P. downsi*		Free-living semi-hematophagous	16	3.2	109.4
*P. niger*	*P. nigra*	Subcutaneous	1	1	0
*P. deceptivus*	*P. deceptiva*	Subcutaneous	6	2.7	95.7
*P. trinitensis*		Subcutaneous	8	2.9	93.6
*P. glaucinis*		Subcutaneous	1	1	0
*P. querulus*	*P. querula*	Subcutaneous	1	1	0
*P. angustifrons*		Subcutaneous	10	3.6	141.5
*P. sanguinis*		Subcutaneous	3	3.7	154.4

### Host bird and parasite phylogenies

**Figure 1:**
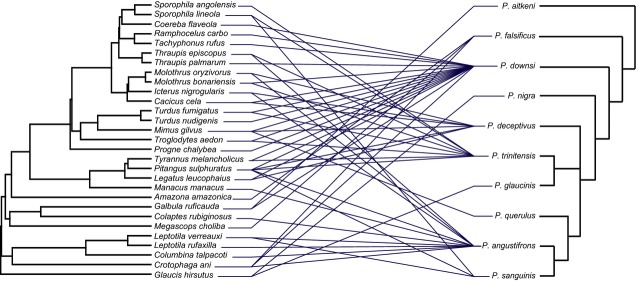
Tanglegram showing the associations between ten *Philornis* species (on the right) and 29 bird host species (on the left) on the island of Trinidad. Thin lines indicate host–parasite associations.

**Figure 2:**
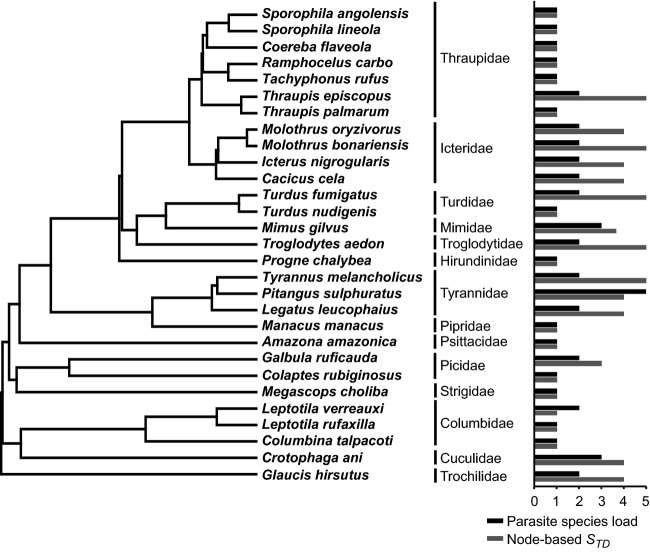
The phylogenetic signal of the birds' parasite species load and node-based *S*_TD_. *Philornis* species were reared from bird species at random in relation to the bird phylogeny.

We generated a phylogeny of the 29 bird species reported as hosts of *Philornis* spp. in Trinidad using the Bayesian pseudo-posterior distribution of time-calibrated bird phylogenies from Jetz et al. (2012) available at the Web site http://birdtree.org. From 5000 trees sampled, we generated a maximum clade credibility (MCC) tree, using the software TreeAnnotator included in BEAST v.1.8.2 (Drummond et al. 2012). This tree was further modified with updated information on Thraupidae (tanagers) from Barker et al. (2015). This bird phylogeny was used in all analyses (Figs.1, 2).

No molecular phylogeny is currently available for the genus *Philornis*. The only available *Philornis* phylogeny is based on morphological characters (Couri et al. 2007). We pruned this phylogeny to include only the ten Trinidadian *Philornis* species and used the software TreeMap v3.0b (Charleston and Robertson 2002) to show the associations between the *Philornis* and the host bird phylogenies (Fig.1).

### Host specificity, parasite species load, and community structure analyses

We recorded the number of bird species exploited by each *Philornis* species and estimated the host specificity index, *S*_TD_, for parasite species with more than one host species. To calculate the index, it is necessary to place each pair of host species of a particular parasite within a Linnaean hierarchy (e.g., class, order, family, genus, and species). The average taxonomic distinctness is the number of steps that must be taken to reach a common ancestor to two host species, averaged across all possible pairs of host species as outlined by Poulin and Mouillot (2003). *S*_TD_ is inversely proportional to specificity; a high index value means that, on average, the hosts of a parasite species are not closely related. We calculated *S*_TD_ using the software TaxoBiodiv2 (Poulin and Mouillot 2005). In addition, we modified the *S*_TD_ index by using the branch length values from the bird phylogeny to estimate a ‘branch length-based *S*_TD_ index’ as an additional host specificity measure. In this test, the average branch length between all pairs of hosts of a given parasite species is computed. We then tested for the phylogenetic signal of host range for the raw number of species exploited, the *S*_TD_ index, and the branch length-based *S*_TD_ index by running the Analysis of Traits (AOT) module in the software Phylocom v.4.2 (Webb et al. 2008). Phylocom uses randomization of traits values across the tips of the phylogeny to determine whether patterns of significant clustering of traits can be detected across the phylogeny.

We also assessed the phylogenetic signal of parasite species load using AOT to determine whether the number of *Philornis* species attacking each host species was significantly clustered on the bird phylogeny. As implemented above for host range, we analyzed both the raw number of species as an indicator of parasite load and a phylogenetically informed measure of parasite species load. Because all parasite species in our study belong in the same genus, we modified the *S*_TD_ index by counting the number of nodes taken to reach a common ancestor between two parasites in the *Philornis* phylogeny, computed across all possible pairs of parasite species attacking each bird species. We called this modified *S*_TD_ index ‘the node-based *S*_TD_’. We then performed the AOT in Phylocom to test for the phylogenetic signal of parasite species load for the raw number of parasite each bird harbored (species richness parasite species load) and the node-based *S*_TD_ index (phylogenetically informed parasite species load).

Lastly, we examined the relationship of the parasite community similarity to the phylogenetic distance between the hosts. We calculated community similarity in the parasite faunas for all possible pairs of host birds using the Jaccard index (Jaccard 1912). This index corresponds to the number of parasite species shared by two host species divided by the total number of parasite species occurring in the two host species; it ranges from 0 (no shared parasite) to 1 (the two bird species have exactly the same parasites) (Poulin 2010). We computed the Jaccard index using the software EstimateS v9.10 (Colwell 2013) and used branch length values to calculate the phylogenetic distance between each of the 406 bird species pairs in this study. Following standard practice, similarity (Jaccard index) and phylogenetic distance measures were log + 1-transformed prior to performing a linear regression between the two variables (Poulin 2010).

## Results

### Host specificity

Four of the ten *Philornis* species were reared from a single host species: *P. aitkeni*, *P. niger*, *P. glaucinis*, and *P. querulus*, and the remaining six species were reared from more than one host bird species (Fig.1). Of these, *P. falsificus* and *P. sanguinis* parasitized three bird species each, *P. deceptivus* six, *P. trinitensis* eight, *P. angustifrons* ten, and *P. downsi* sixteen (Table1). *Philornis falsificus*, with the highest *S*_TD_ value, parasitizes three bird species in three different orders; therefore, this is the least specialized parasite species when host phylogeny is taken into account, whereas *P. deceptivus* exploits six bird species, all in the Passeriformes, being the most host specific of the *Philornis* species that has been reared from more than one host species (Table1).

We found no evidence that host specificity is clustered on the *Philornis* phylogeny (phylogenetic signal analyses in Phylocom: *P *=* *0.88 for the raw number of hosts, *P *=* *0.68 for *S*_TD_, *P *=* *0.65 for branch length-based *S*_TD_) or evenly distributed on the phylogeny (*P *=* *0.11 for the raw number of hosts, *P *=* *0.31 for *S*_TD_, *P *=* *0.35 for branch length-based *S*_TD_).

### Parasite species load

The number of parasite species harbored per bird species also varied, with most birds hosting one or two *Philornis* species but one bird (the great kiskadee, *Pitangus sulphuratus*) serving as host to five *Philornis* species (Fig.1). The analysis of phylogenetic signal of parasite species load on the host phylogeny showed no significant effect for parasite species richness or the node-based *S*_TD_ (Fig.2; *P *=* *0.29 for the parasite species load, *P *=* *0.57 for the node-based *S*_TD_).

### Community structure

**Figure 3:**
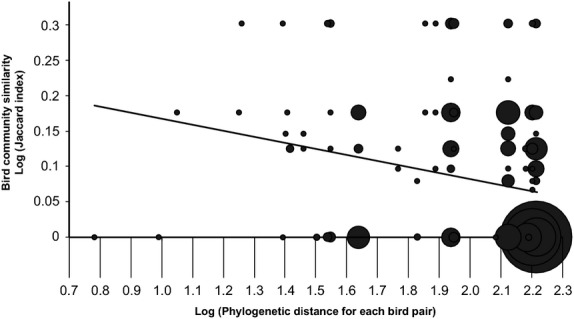
Parasite community similarity as a function of the phylogenetic distance between host birds, in log–log space. Phylogenetic distance is expressed as the total branch length separating each bird pair. The area of the circles is proportional to the number of observations.

We found a significant decrease in the community similarity of host species attacked by *Philornis* species (measured by the Jaccard index) as a function of increasing phylogenetic distance between the host birds (Fig.3). The significant linear regression (*r*^2^ = 0.05, *P *<* *0.0001) of log community similarity against log phylogenetic distance suggests that *Philornis* species on Trinidad tend to feed on closely related birds more often than on distantly related bird species.

## Discussion

The *Philornis* fauna in Trinidad contains both specialists and generalists*,* with varying degrees of specificity within the generalists, and this general pattern agrees with an analysis of *Philornis* spp. found outside of Trinidad (Löwenberg-Neto 2008). Four *Philornis* species were only reared from one bird species in Trinidad. These are *P. aitkeni*, *P. niger*, *P. querulus,* and *P. glaucinis*. The number of bird species attacked by the remaining six *Philornis* species ranged from three (*P. falsificus* and *P. sanguinis*) to sixteen (*P. downsi*). The host associations of *Philornis* spp. in Trinidad included seven bird orders, as compared to ten known throughout its distributional range (Teixeira 1999; Dudaniec and Kleindorfer 2006).

Comparing three measures of host specificity – species richness, the *S*_TD_ index, and the branch length-based *S*_TD_ – showed the usefulness of including phylogenetic information to assess host specificity (Poulin and Mouillot 2003; Poulin et al. 2011). In particular, the two *Philornis* species that were reared from only three host species had the highest *S*_TD_ values, indicating a broad phylogenetic host range. This is because these three host species belonged to three different bird orders (in the case of *P. falsificus*) or two host orders with divergent taxa within the common orders (in the case of *P. sanguinis*) (see Table1 and Fig.1). The four other *Philornis* species that were reared from between six and sixteen host species attacked birds from within a narrower phylogenetic breadth of hosts. The most specialized of these, *P. deceptivus*, was reared from six species of birds, all within the order Passeriformes.

The larval feeding habit of *Philornis* species may have an influence on the host range, but we have insufficient data to evaluate this formally. Coprophagy, which is considered the ancestral feeding habit for *Philornis* (Couri et al. 2007; but see Dodge 1971), is represented by a single species in our data set (*P. aitkeni*) which was reared from a single bird species (the rufous-tailed jacamar, *Galbula ruficauda*). There is no other information range-wide on host associations of this species, but it is interesting that the only other coprophagous *Philornis* species, *P. rufoscutellaris*, is also only known from *G. ruficauda* (Teixeira 1999). Thus, coprophagy may be linked to specialization in *Philornis*. Of the truly parasitic species, both of the free-living semi-hematophagous species (*P. downsi* and *P. falsificus*) exhibited a broad host range (when measured by the number of host species or the *S*_TD_) while the subcutaneous species had widely varying host ranges (Table1). Free-living semi-hematophagy is considered an evolutionary transition toward subcutaneous feeding (Couri et al. 2007), and the broad patterns exhibited by *Philornis* in Trinidad are consistent with the hypothesis that this transition is associated with host-range restriction in some cases.

Despite these trends, we found no significant statistical effect of the *Philornis* phylogeny on host range, whether it was measured as species richness or the *S*_TD_ index. This is somewhat unexpected as the ability to recognize and successfully exploit hosts is presumed to be under strong selection (Poulin 2007) and thus could lead to lineages with similar host associations. However, various models of speciation involve changes in host specificity. For instance, a generalist lineage may give rise to one or more specialized lineages (Moran 1988) and vice versa (Stireman 2005). Either of these cases would interfere with a phylogenetic signal of host specificity. As we noted above, the most ancestral parasite species in our data set is the specialist *P. aitkeni*, and its nearest relatives have a very broad host range (Fig.1). This suggests host-range expansion within Trinidad over the timescale that these lineages evolved. A possible case of host-range constriction involves the specialist *P. querulus*, which was reared only from the tropical mockingbird, *Mimus gilvus*, while its closest known ancestor *P. deceptivus* was reared from six hosts, including *M. gilvus*. Thus, it appears that the evolutionary history of *Philornis* may involve a mosaic of host-range expansion and contraction resulting in no clear clustering of host range on the phylogeny.

We also did not find a significant effect of the host phylogeny on the parasite species load. Despite this, two bird families did show consistently high values of the node-based *S*_TD_ index (Fig.2): the blackbirds and relatives (Icteridae) and the flycatchers (Tyrannidae). These birds might have a higher level of susceptibility to parasitism by *Philornis,* or there may be ecological factors (such as abundance) that make them particularly vulnerable.

Finally, we performed a phylogenetically informed analysis of community structure to determine whether *Philornis* tend to attack birds that are themselves closely related. This is illustrative of a trend to incorporate phylogenetic information into analyses of community structure (Cavender-Bares et al. 2009). We found that *Philornis* species do indeed tend to parasitize bird species that are more closely related than expected by chance. This is in agreement with the literature on parasite faunas in general (Poulin 2010), but we note that the trend is relatively weak (Fig.3).

A number of difficulties can arise when studying host specificity from published records in the literature (reviewed by Poulin 2007). First, sampling effort can account for much of the variability in the number of known host species. Second, misidentification of parasite or host species can compromise the estimate of host specificity. We note that in the study analyzed here, a substantial amount of time was spent collecting (the sampling period spans 6 years) and the ten *Philornis* species were presumably correctly identified by the authors, who were themselves the taxonomic authorities on the genus *Philornis*. However, a more useful index of host specificity would include information on the abundance or prevalence of parasitism (Poulin et al. 2011).

The data on host associations of *Philornis* species in locations other than Trinidad are rather sparse (Teixeira 1999; Dudaniec and Kleindorfer 2006; Löwenberg-Neto 2008). For some species, however, a comparison between host associations found on Trinidad and in other locations can be made. For example, *P. glaucinis* has been reared from only one bird species each in Trinidad and Panama (the rufous-breasted hermit, *Glaucis hirsutus,* and the crimson-backed tanager, *Ramphocelus dimidiatus*, respectively) (Teixeira 1999; Bermúdez et al. 2010) while in Brazil it is recorded from species belonging to eight bird families, including two species that are also attacked in Trinidad (the shiny cowbird, *Molothrus bonariensis,* and the tropical screech owl, *Megascops choliba*) (Teixeira 1999). The intensive sampling in Trinidad suggests true specificity there at least (the case for specificity in Panama is less clear), but as the geographic origin of *P. glaucinis* is not known, it is not clear whether these differences reflect increasing host specialization in Trinidad or host-range expansion in Brazil. The island of Trinidad is thought to have separated from the South American mainland approximately 1500 years ago (Kenny 2008), so there was likely little physical isolation of the faunas over evolutionary time.

Another interesting case is *Philornis downsi*, which is known from a number of localities in mainland South America, and has recently invaded the Galápagos Islands. This species occurs in Argentina (Silvestri et al. 2011), Brazil (Couri 1984, 1999), and mainland Ecuador (Bulgarella et al. 2015). After its accidental introduction into the Galápagos Islands, it has been reported parasitizing 18 species of birds in five families (Fessl and Tebbich 2002; O'Connor et al. 2010; Causton et al. 2013), including 14 endemic species that represent a clear expansion of host range for this fly. In Trinidad, *P. downsi* was the *Philornis* species that parasitized the highest number of host birds (16); in Ecuador, *P. downsi* also appears to be the dominant species of *Philornis* (Bulgarella et al. 2015) and has been reared from more bird species than other *Philornis* spp. (M. Bulgarella, M. A. Quiroga, G. A. Brito Vera, and G. E. Heimpel, unpublished). We suggest that the broad host range of this species in its native range may have contributed to its invasiveness in the Galápagos Islands.

## Conclusion

Information on host–parasite associations can suggest evolutionary patterns such as host-range expansion or contraction or the co-evolution of host and parasite lineages. Hypotheses such as these can be best addressed when phylogenies are available for the hosts and/or the parasites. Here we have used this approach to explore host associations between birds and their *Philornis* parasites on the island of Trinidad. No clear trends were uncovered with respect to a phylogenetic pattern of host range or parasite species load, but we found that individual *Philornis* species were likely to attack bird species that were relatively closely related.

## Supporting Information

**Table S1.** Twenty-nine bird species host to ten *Philornis* species in the Island of Trinidad.
