# Supplementary material for: Host range and community structure of avian nest parasites in the genus Philornis (Diptera: Muscidae) on the island of Trinidad
Source: Ecol Evol. 2015 Aug 15;5(17):3695–703. doi: 10.1002/ece3.1621 (PMC4567873; doi:10.1002/ece3.1621)
Supplement: Supplementary file 1 — Table S1. Twenty-nine bird species host to ten Philornis species in the Island of Trinidad. [file ece30005-3695-sd1.docx]

**Table S1.** Twenty-nine bird species host to ten *Philornis* species in the Island of Trinidad. Taxonomic classification follows the Cornell University Neotropical Birds website (http://neotropical.birds.cornell.edu/). Between parentheses is the species name as listed in the Dodge and Aitken (1968) paper.

| **Species name** | **Bird Order** | **Bird Family** | **How many *Philornis* spp. does it harbor?** | **Nest type** |
| --- | --- | --- | --- | --- |
| *Amazona amazonica* | Psittaciformes | Psittacidae | 1 | Cavities |
| *Cacicus cela* | Passeriformes | Icteridae | 2 | Enclosed, pouch-like, hanging nest |
| *Coereba flaveola* | Passeriformes | Thraupidae | 1 | A compact globe with round doorway facing obliquely downward |
| *Colaptes (= Piculus) rubiginosus* | Piciformes | Picidae | 1 | Hole nesting |
| *Columbina (=Columbigallina) talpacoti* | Columbiformes | Columbidae | 1 | Shallow cup nest |
| *Crotophaga ani* | Cuculiformes | Cuculidae | 3 | Deep cup nest, built communally by several pairs |
| *Galbula ruficauda* | Piciformes | Picidae | 2 | Burrow |
| *Glaucis hirsutus (=hirsuta)* | Apodiformes | Trochilidae | 2 | Small cup nest with a tail |
| *Icterus nigrogularis* | Passeriformes | Icteridae | 2 | Pendant |
| *Legatus leucophaius* | Passeriformes | Tyrannidae | 2 | It does not build its own nest, but appropriates the domed or enclosed nests of other bird species |
| *Leptotila rufaxilla* | Columbiformes | Columbidae | 1 | Large stick nest in a bush or on a stump |
| *Leptotila verreauxi* | Columbiformes | Columbidae | 2 | Large stick nest in a tree |
| *Manacus manacus* | Passeriformes | Pipridae | 1 | Cup |
| *Megascops (=Otus) choliba* | Strigiformes | Strigidae | 1 | Cavities |
| *Mimus gilvus* | Passeriformes | Mimidae | 3 | Cup |
| *Molothrus bonariensis* | Passeriformes | Icteridae | 2 | Brood parasite (it does not build its own nest) |
| *Molothrus (= Psomocolax) oryzivorus* | Passeriformes | Icteridae | 2 | Brood parasite (it does not build its own nest) |
| *Pitangus sulphuratus* | Passeriformes | Tyrannidae | 5 | A dome structure, built in an exposed tree branch. Open cup nests have been observed, placed in tree cavities or concealed places |
| *Progne chalybea* | Passeriformes | Hirundinidae | 1 | Secundary cavity nesting |
| *Ramphocelus carbo* | Passeriformes | Thraupidae | 1 | Cup |
| *Sporophila (=Oryzoborus) angolensis* | Passeriformes | Thraupidae | 1 | Cup |
| *Sporophila lineola* | Passeriformes | Thraupidae | 1 | Cup |
| *Tachyphonus rufus* | Passeriformes | Thraupidae | 1 | Cup |
| *Thraupis episcopus (= virens)* | Passeriformes | Thraupidae | 2 | Deep cup |
| *Thraupis palmarum* | Passeriformes | Thraupidae | 1 | Cup |
| *Troglodytes aedon (= musculus)* | Passeriformes | Troglodytidae | 2 | Cup |
| *Turdus fumigatus* | Passeriformes | Turdidae | 2 | Cup |
| *Turdus nudigenis* | Passeriformes | Turdidae | 1 | Cup |
| *Tyrannus melancholicus* | Passeriformes | Tyrannidae | 2 | Cup |
|  |  |  |  |  |
